# Stools from a human APOEe2 donor reduces amyloid and tau pathology and increases neuroinflammation in a 3xTg AD mouse model

**DOI:** 10.3389/fnagi.2025.1539067

**Published:** 2025-02-14

**Authors:** Moira Marizzoni, Benjamin B. Tournier, Claire Chevalier, Samantha Saleri, Aurélien Lathuilière, Kelly Ceyzériat, Arthur Paquis, Rahel Park, Emma Troesch, Annamaria Cattaneo, Philippe Millet, Giovanni B. Frisoni

**Affiliations:** ^1^Biological Psychiatry Unit, IRCCS Istituto Centro San Giovanni di Dio Fatebenefratelli, Brescia, Italy; ^2^Department of Psychiatry, University Hospitals of Geneva, Geneva, Switzerland; ^3^Department of Psychiatry, University of Geneva, Geneva, Switzerland; ^4^Memory Clinic and LANVIE-Laboratory of Neuroimaging of Aging, University Hospitals and University of Geneva, Geneva, Switzerland; ^5^CIBM Center for Biomedical Imaging, Geneva, Switzerland; ^6^Department of Pharmacological and Biomolecular Sciences, University of Milan, Milan, Italy; ^7^Geneva Memory Center, Department of Rehabilitation and Geriatrics, Geneva University Hospitals, Geneva, Switzerland

**Keywords:** Alzheimer’s disease pathology, microbiota, transplantation, APOEe2, human donor

## Abstract

**Background:**

The mechanisms underlying the protective effect of the e2 variant of the APOE gene (APOEe2) against Alzheimer’s disease (AD) have not been elucidated. We altered the microbiota of 3xTgAD mice by fecal microbiota transplantation from a human APOEe2 donor (e2-FMT) and tested the effect of microbiota perturbations on brain AD pathology.

**Methods:**

FMT of bacteria isolated from stools of untreated 3xTgAD mice (M-FMT) or e2-FMT were transplanted in 15-month-old 3xTgAD mice. FMT was done alone or in combination with antibiotic and proton-pump inhibitor following the Microbiota Transfer Therapy protocol (MTT). The effect of donor (M or e2) and transplantation protocol (FMT or MTT) on hippocampal amyloid, tau pathology and neuroinflammation were assessed at the end of the treatment.

**Results:**

e2-FMT reduced amyloid, and tau pathology as well as increased neuroinflammation as compared with M-FMT. MTT was associated with reduced number of Aβ40+ plaques and tau pathology. Low levels of amyloid were associated with high levels of pro-inflammatory molecules in e2-FMT mice. These associations were partially attenuated by MTT.

**Conclusion:**

Bacteria from a human APOEe2 donor reduced AD pathology and increased neuroinflammation in mice suggesting that the gut microbiota may be a mediator of the protective effect of APOEe2.

## Background

The e2 allele of the APOE gene (APOEe2) is associated with a lower risk of developing Alzheimer’s disease (AD) but the mechanisms underlying its influence on the onset and progression of AD have not been elucidated. Evidence suggests that APOEe2 protects against AD through amyloid beta (Aβ)-dependent mechanisms. APOEe2 carriers have lower Aβ burden among non-demented aged individuals and AD patients (Reiman et al., 2020; Serrano-Pozo et al., 2015) and, among individuals with minimal Aβ pathology, APOEe2 carriers are more likely cognitively intact compared with APOEe3 (Shinohara et al., 2016). Furthermore, apoEe2 protein abundance in human postmortem cortex follows an isoform-dependent pattern (e2 > e3 > e4) and, coupled with apoEe2 inability to bind to low density lipoproteins, might increase amyloid-beta (Aβ) clearance (Conejero-Goldberg et al., 2014). However, several studies propose that the e2 allele might reduce the AD risk also through Aβ-independent pathways. For example, APOEe2 confers larger gray matter volumes in brain areas relevant for AD (Salvadó et al., 2021) and greater cortical thicknesses in healthy elderly adults (Fan et al., 2010).

APOE has physiological effects not restricted to the brain including the modulation of the immune system and gut microbiota (GM) composition. Previous research reports conflicting results on the effect of E2 on inflammatory markers with human studies showing a pro-inflammatory effect (Civeira-Marín et al., 2022; Hubacek et al., 2010; Schick et al., 2015) and *in vitro* studies reporting an anti-inflammatory effect (de Leeuw et al., 2022). As for the central nervous system, the impact of the e2 allele on the microbiota has received much less attention than the e4 variant. While many of the studies show the deleterious effect of APOEe4 on the composition of the microbiota (i.e., association with pathogens belonging to Proteobacteria in AD patients) (Hou et al., 2021) or with loss of butyrate-producing bacteria in cognitively healthy APOEe4 carriers (Tam et al., 2019), data on APOEe2 are still scarce.

Several strategies to modulate gut microbiota in preclinical models of AD have been proposed, including fecal microbiota transplantation (FMT) and antibiotics (Bello-Medina et al., 2022; Dodiya et al., 2019; Elangovan et al., 2023; Sun et al., 2019). Most of the studies have investigated the effect of the transfer of material from mouse to mouse and, to the best of our knowledge, none have investigated the impact of human fecal microbiota in mouse models of AD. From a clinical perspective, a FMT protocol including the administration of antibiotics and a proton-pump inhibitor (PPI), called Microbiota Transfer Therapy (MTT), has shown both a short-term and long-term beneficial effect on clinical symptoms and microbiota composition in patients with autism (Kang et al., 2017, 2019).

To test the hypothesis that the protective effect of the e2 allele in AD is mediated in part by the gut microbiota, we altered the microbiota of 3xTgAD mice using fecal microbiota transplantation from a human APOEe2 donor (e2-FMT), administered alone or applying the MTT protocol, and tested the effect on hippocampal AD pathology.

## Methods

### Study design and experimental timeline

Thirty 15-month-old female 3xTgAD mice (APPSWE, PS1M146V and TauP301L), bred in a conventional facility at the University of Geneva, were used for the FMT treatment. All animals were reared under standard light/dark conditions with ad libitum access to food and water. Animals were group-housed (2–3 per cage) and randomly assigned in 2 groups. One group received the FMT treatment alone, where an initial high dose of fecal microbiota transplantation (FMT) (10^9^cells/100 μL) was followed by a low dose of FMT (10^6^cells/100 μL) once per week for 2 months. In the second group, a treatment with antibiotics and PPI (Kang et al., 2017) was added to FMT (Supplementary Figure 1A). In particular: (i) antibiotic treatment for 14 days in the drinking water (100 μg/mL Ampicillin, 100 μg/mL Cefoperazone Sodium Salt, 100 μg/mL Clindamycin Hydrochloride), renewed 3 times per week; (ii) laxative administration (Moviprep 30 mg/kg), 24 h before the first FMT; (iii) FMT as described above; and (iv) stomach acid suppressant (the proton-pump inhibitor Omeprazol, 30 mg/in 100 mg Madidrop Sucralose) once every working day. After 2 months of treatment, mice were sacrificed under anesthesia after intracardiac saline perfusion. Euthanasia was performed under isoflurane anesthesia (3%) by bleeding following incision of the right atrium and perfusion of the left ventricle with 0.9% NaCl solution, after opening the thoracic cavity. The brain was collected, and one hemisphere was immersed in formalin for 24 h and then embedded in paraffin for carrying out immunohistochemistry and immunofluorescence. The other hemisphere was dissected on ice to isolate the hippocampus for protein measurement. The samples were frozen in liquid nitrogen and then stored at −80°C.

### Fecal microbiota transplantation preparation

A participant with normal cognition (Mini-Mental state examination score equal to 28), 72 years old, APOE e2/e3 genotype and without brain amyloidosis (PET image visually scored as negative for pathologic levels of Aβ aggregation by a trained nuclear medicine physician) was recruited at the Geneva Memory center within the gMAD/COSCODE study. The donor provided fresh stools in a Feconcontener® that were processed within 2 h from collection. For the mouse FMT, stools from 24 h were collected in the cages of the untreated 3xTgAD mice before the initiation of the experiment. Untreated mice were chosen as controls instead of wild type mice to test the effect of the FMT technique on AD markers and to be certain that the changes in amyloid and tau levels were associated with the treatment (APOEe2 microbiota) and not the route of FMT administration. The human and the mouse fecal material was entirely processed under anaerobic conditions using an anaerobic chamber. In particular, 50 g of stool sample was homogenized in 250 mL of sterile anaerobic PBS and filtered through 2, 1, 0.5 and 0.25 mm sieves. The resulting material was centrifuged at 6,000 × *g* for 15 min and resuspended in 20 mL of sterile anaerobic PBS. Living bacteria were counted with the live/dead backlight bacterial viability and counting kit (Invitrogen, Catalog # L34856) and diluted to 10^9^cells/100 μL and 10^6^cells/100 μL in sterile anaerobic PBS 10% glycerol before aliquoting and freezing at −80°C until further use. FMT was administered by gavage with a sterile plastic cannula of 100 μL of the indicated preparation thaw 1 h before use.

### ELISA tests

The hippocampus of one hemisphere was sonicated in a solution of triton (50 mM Tris HCl, 150 mM NaCl, 1%Triton x100 (Tx), protease and phosphatase inhibitors 1x, pH = 7.4) and centrifuged (20,000 *g*, 20 min, 4°C). The supernatants collected from the triton soluble fraction was used for ELISA tests. In addition, for the quantification of highly aggregated Aβ42 forms, the pellet was dissolved in a guanidine buffer (5 M guanidine (Gua), 50 mM Tris HCl, protease and phosphatase inhibitors 1x, pH = 8), gently agitated (3 h, 4°C), centrifuged (20,000 *g*, 20 min, 4°C) and the guanidine soluble fraction in the supernatant was collected. A protein assay was performed by BCA to express the data measured by ELISA as a function of the total amount of proteins. ELISA detection of proteins was performed using manufactured kits and according to the instructions of joined protocols. The kits were: Aβ42 human ultrasensitive ELISA Kit (ThermoFisher Scientific), Aβ40 human ELISA Kit (Thermofisher Scientific), BACE1 ELISA kit (Biotechne) and IDE ELISA kit (Labforce). The OD reading was performed at 450 nm in the presence of a standard curve, specific to each kit. For neuroinflammatory protein quantification, a Luminex multiplex assay kit (Cytokine & chemokine 20-plex mouse ProcartaPlexTM Panel, ThermoFisher Scientific) was used. Among the 20 markers, 3 were beyond the background noise (IFN γ, IL2, IL5) and 6 were excluded from further statistical analysis since more than 20% of the samples were below the limit of detection (IL4, IL13, IL18, MIP1, MIP2, Rantes). BACE1, IDE, TSPO and Luminex multiplex assay kits were only performed on triton-solubilized proteins.

### Immunohistochemistry and immunofluorescence

Brain sections of one hemisphere were used and cut on a microtome (10 μm). After dewaxing, rehydratation, and an antigen retrieval procedure (95°C, 20 min, concentration Tris-Citrate buffer), sections were incubated in 1xPBS, 0.2%Triton X-100 before adding the primary antibody for 48 h incubation at 4°C. Primary antibodies were: 4G8 (Biolegend 800,710, 1/500), Aβ40 (Thermofisher 44–136, 1/300), AT8 (Thermofisher MN1020, 1/500). After 1xPBS washing (2× 5 min), the secondary antibody was added for 90 min at RT before an additional 1xPBS washing (2× 5 min).

### Microbiota analysis

Bacterial DNA was extracted with MagPurix Bacterial DNA Extraction kit using automated Zinexts system, according to the manufacturer’s instructions. For the FMT preparations, 1 mL of 10^9^cell/100 μL preparation was spined and resuspended in 1 mL of RLT buffer. For mouse tissues, 50 mg of frozen cecum content were mechanically disrupted in 1 mL of RLT buffer with bead-beating using Precellys soil grinding kit SK38, 3×40 sec at 6000 rpm, followed by 5 min at 95°C shaking at 600 rpm. DNA was quantified using a NanoDrop ND-1000 spectrophotometer, and then stored at −20°C until subsequent analyses. The regions V3 and V4 of the bacterial 16S sub-unit of the ribosomal RNA 16S (16S rRNA) gene was amplified and purified according to 16S Metagenomic Sequencing Library Preparation protocol by Illumina. The amplicons were pooled in equal concentration and sequenced with Illumina MiSeq PE300. The raw 16S rRNA gene data were processed using QIIME2 (Bolyen et al., 2019) (64 bit version 2023.5) run on a Linux workstation (Ubuntu 20.04.6 LTS) equipped with Intel CPU 32 × 64 GHz processors and 125.6 GB of RAM. Sequencing data were already demultiplexed. Forward and reverse primers, reads containing ambiguous bases or homopolymers greater than eight base pairs in length were removed. Moreover, we set a maximum number of expected errors equal to 2 and reads truncation if the quality score was less than 2. The DADA2 denoising process was applied with the default parameters (Callahan et al., 2016). Alpha diversity, indicating the richness and abundance of ASVs within each individual, and beta diversity, showing the similarity or difference in microbiota composition between individuals, were calculated using the q2-diversity plugin after rarefaction of the feature table at the sample depth of 18,317 (sample depth corresponding to the sample with the lowest read count). Alpha and Beta diversity included Bray-Curtis and Jaccard indexes, Shannon and Pielou’s evenness indexes. SILVA reference database (version 138),^1^ customized following the instructions on the dedicated tutorials and as previously reported (Marizzoni et al., 2020), was used to infer the taxonomy of the amplicon sequence variants (ASV) at phylum and genus level. Absolute abundances at the genus and phylum levels were normalized by the total number of reads assigned in each sample. Genera with incomplete taxonomy, assigned to the Eukaryota domain or found with relative abundance lower than 10^−3^ across all samples were removed. A total of 130 ASVs were identified in the whole group after filtering.

### Statistical analysis

Statistical analyses were performed using R (v.4.3.0) with the Rstudio (2023.03.1 + 446), unless specified otherwise. The effect of donor selection (M or e2), transplantation protocol (FMT alone or MTT) and their interaction on microbiota and brain variables was assessed using two-way ANOVA with Tukey test for multiple comparisons. Values that were above or below 2.5 standard deviations from the group mean were excluded from the analysis. Beta diversity was analyzed using permutational multivariate analysis of variance [PERMANOVA, R packages: vegan v2.6–4 (Oksanen et al., 2012)], and differential abundance of microbial genera using the Wilcoxon signed-rank test implementation in the ALDEx2 library (Fernandes et al., 2013). Significant differentially abundant genera between pairs of treatments were selected according to the effect sizes higher than 1 or lower than −1. Three-dimensional principal coordinate analysis (PCoA) was used to visualize beta diversity results. All figures were generated using ggplot2 (Wickham, 2016). Associations of inflammatory variables with amyloid and tau markers were assessed with Spearman correlation, the nonparametric version of the Pearson correlation which reduces the influence of extreme values. Significance was set at *p* < 0.05 (two-tailed).

## Results

### FMT from the APOEe2 donor and MTT modulated the GM composition in 3xTg mice

**Figure 1:**
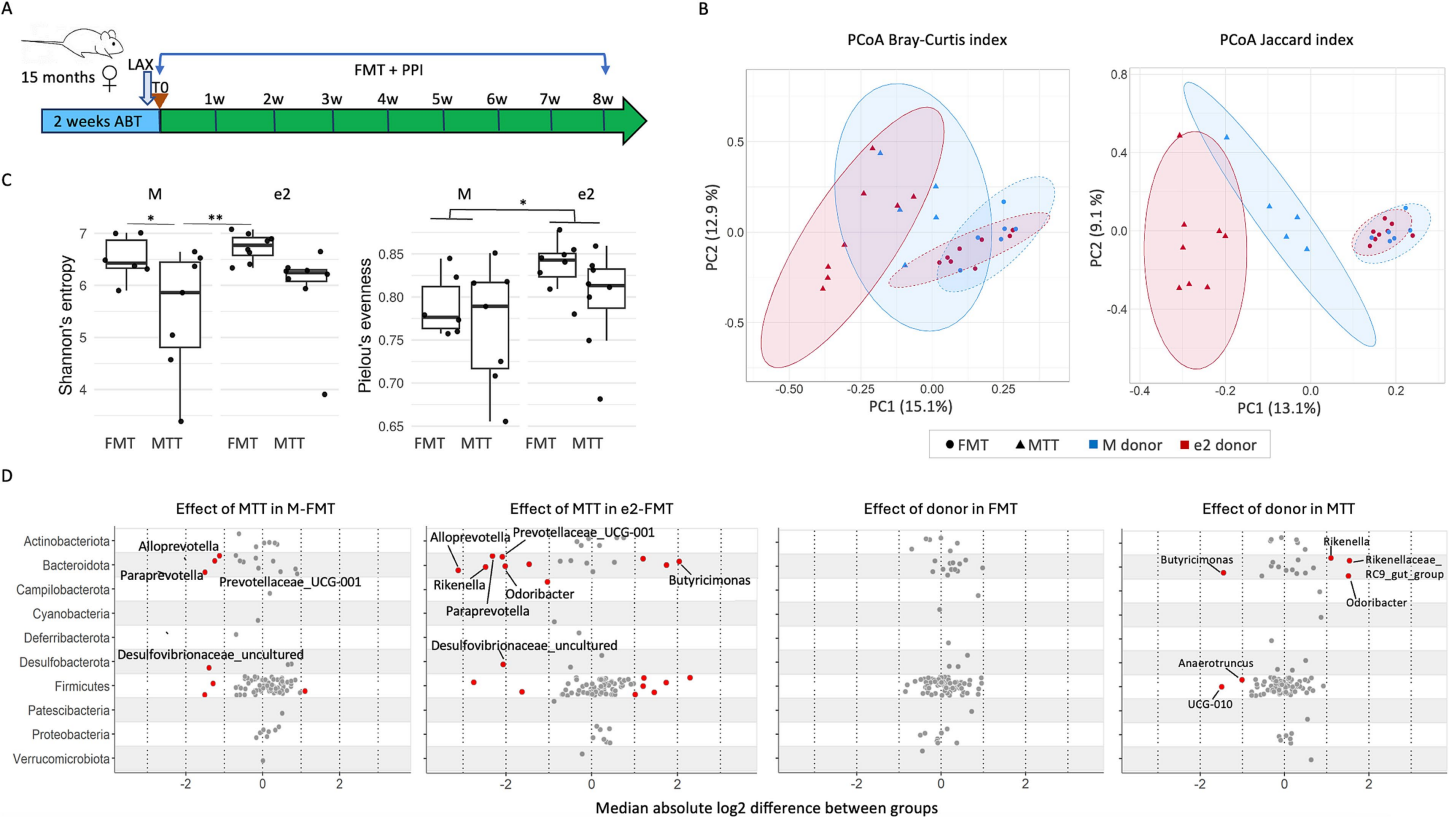
Effect of donor (M or e2) and transplantation protocol (FMT or MTT) on bacteria community. **(A)** Study design; **(B)** Principal coordinate analysis (PCoA) showing the effects of donor and transplantation protocol on β-diversity (as measured by Bray-Curtis and Jaccard distance). Ellipses indicate 95% confidence intervals per group; **(C)** Box plots showing alpha diversity of mice as measured by Pielou’s evenness and Shannon indexes; **(D)** Genera altered by donor and transplantation protocol. Wilcoxon signed-rank test followed by Benjamini–Hochberg correction as per the ALDEx2 library. Color denotes effect size, with red indicating genera significantly different between groups as defined by absolute effect size equal or higher than 1. FMT, fecal microbiota transplantation alone; LAX, laxative; MTT, microbiota transfer therapy consisting of FMT plus antibiotics plus PPI treatment; e2, mice treated with bacteria isolated from an aged human APOEe2; M, mice treated with bacteria isolated from an untreated 3xTg mouse; PPI, proton pump inhibitor. *p < 0.05; **p < 0.01.

To explore the influence of the APOEe2 genotype on Alzheimer’s pathology, bacteria isolated from stools of a cognitively healthy subject, carrier of an APOEe2 allele and without amyloidosis were transplanted into 15-month-old 3xTg female mice. The impact of antibiotics and PPI administration (MTT) on AD pathology was also assessed (Figure 1A).

To evaluate whether hippocampal changes were associated with the composition of the engrafted GM, we analyzed the cecal content composition with 16S rRNA gene sequencing. Principal coordinate analysis (PCoA) of the Bray-Curtis and Jaccard distance matrix revealed that the microbial structure was influenced by transplantation protocol (*F* > 2.30, *p* < 0.018; Figure 1B and Supplementary Table 1). Notably, a trend was reported for the interaction term (*F* = 1.81, *p* = 0.053 for BC; *F* = 1.60, *p* = 0.083 for JC) and M- and e2-recipient mice clustered together when treated with FMT but not with MTT. A significant effect of donor selection and transplantation protocol was observed also for alpha measures, mainly for Shannon’s index (*F* > 4.89, *p* < 0.037; Figure 1C and Supplementary Table 2). The decrease in alpha diversity metrics detected in the MTT groups suggested a lower diversity of bacterial species in these mice. In line with β- and α-diversity analyses, the strongest effect on genera abundance was reported for the MTT groups, regardless of the donor (Figure 1D). The genera most affected by the MTT were *Alloprevotella*, *Prevotellaceae_UCG-001*, *Paraprevotella* and an uncultered genus belonging to the *Desulfovibrionaceae* family (ALDEx2 effect size≥1, Figure 1D). An effect of the donor was reported for *Butyricimona, Rikenella*, *Rikenellaceae_RC9_gut_group*, *Odoribacter*, *Anaerotruncus* and *UCG-010* but only in mice that received MTT. When only FMT was administered, no difference related to donors was reported.

### FMT from the APOEe2 donor reduced amyloid and tau proteins in the whole hippocampus

**Figure 2:**
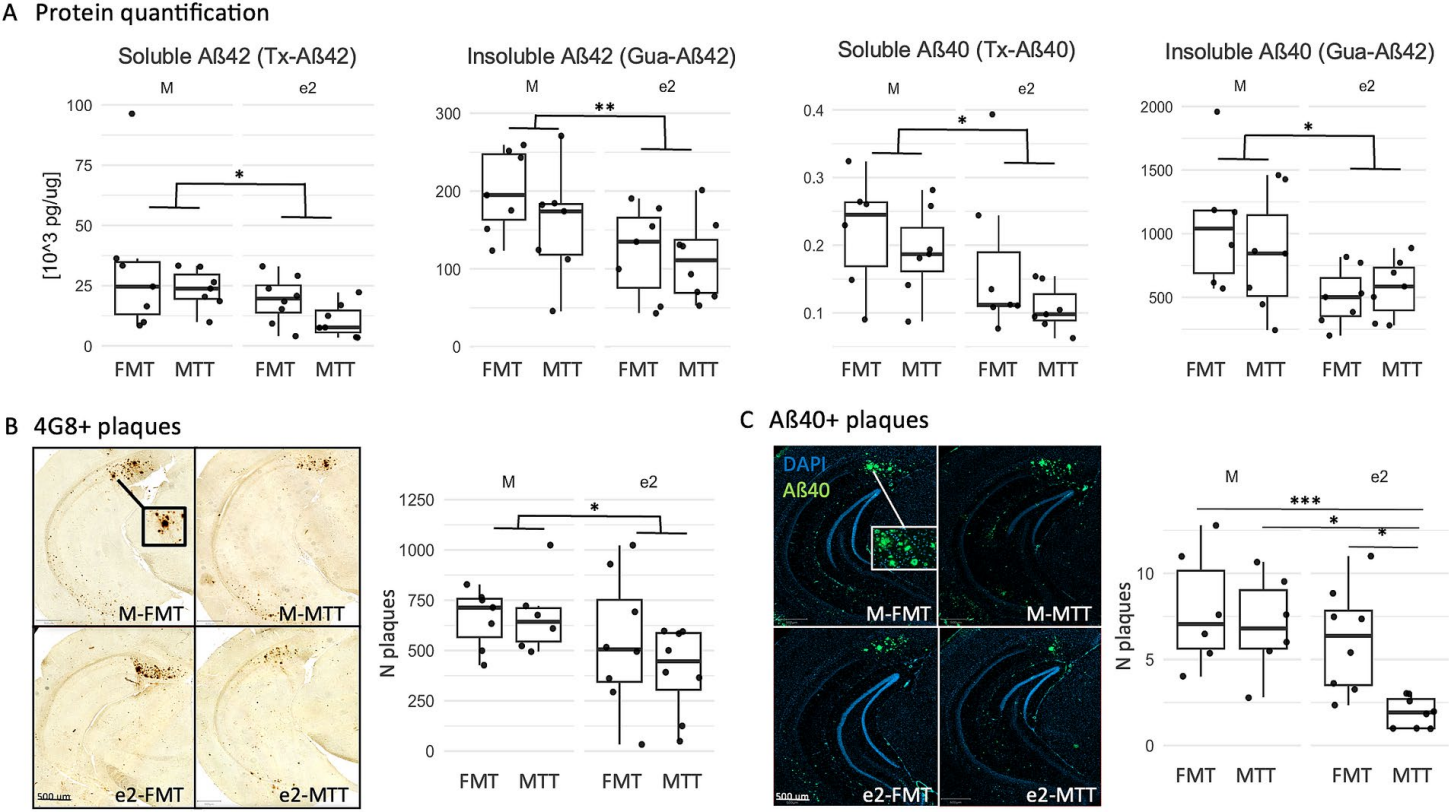
Effect of FMT from the APOEe2 carrier donor and MTT on decreasing amyloid. **(A)** Soluble and insoluble protein quantification of Aβ burden was performed using ELISA in the whole hippocampus. **(B)** Representative images of 4G8 **(B)** and Aβ40 **(C)** positive plaques in the subiculum and their number. Data are presented as boxplots with black horizontal lines indicating medians and circles indicating the subject’s values. Differences in means were tested by two-way ANOVA with donor (M or e2), transplantation protocol (FMT or MTT) and their interaction as factors and with Tukey test for multiple comparisons. *p < 0.05; **p < 0.01; ***p < 0.001. FMT, fecal microbiota transplantation alone; MTT, microbiota transfer therapy consisting of FMT plus antibiotics plus PPI treatment; e2, mice treated with bacteria isolated from an aged human APOEe2; M, mice treated with bacteria isolated from an untreated 3xTg mouse.

An effect of the donor but not of MTT was observed on the levels of soluble and poorly aggregated forms (Tx-Aβ) and highly aggregated forms (Gua-Aβ) insoluble Aβ42 and Aβ40 as well as on 4G8 positive plaques (*F* > 4.27, *p* < 0.049; Figures 2A,B and Supplementary Table 2). Moreover, an additive effect of MTT on FMT emerged in the e2 treated mice on the number of Aβ40 positive plaques (e2-FMT vs. e2-MTT, *p* = 0.037; Figure 2C and Supplementary Table 2). Finally, no effect of donor selection and transplantation protocol on enzymes involved in the amyloid synthesis (BACE1) and degradation (IDE) was reported (*p* > 0.079; Supplementary Figure 1 and Supplementary Table 1).

**Figure 3:**
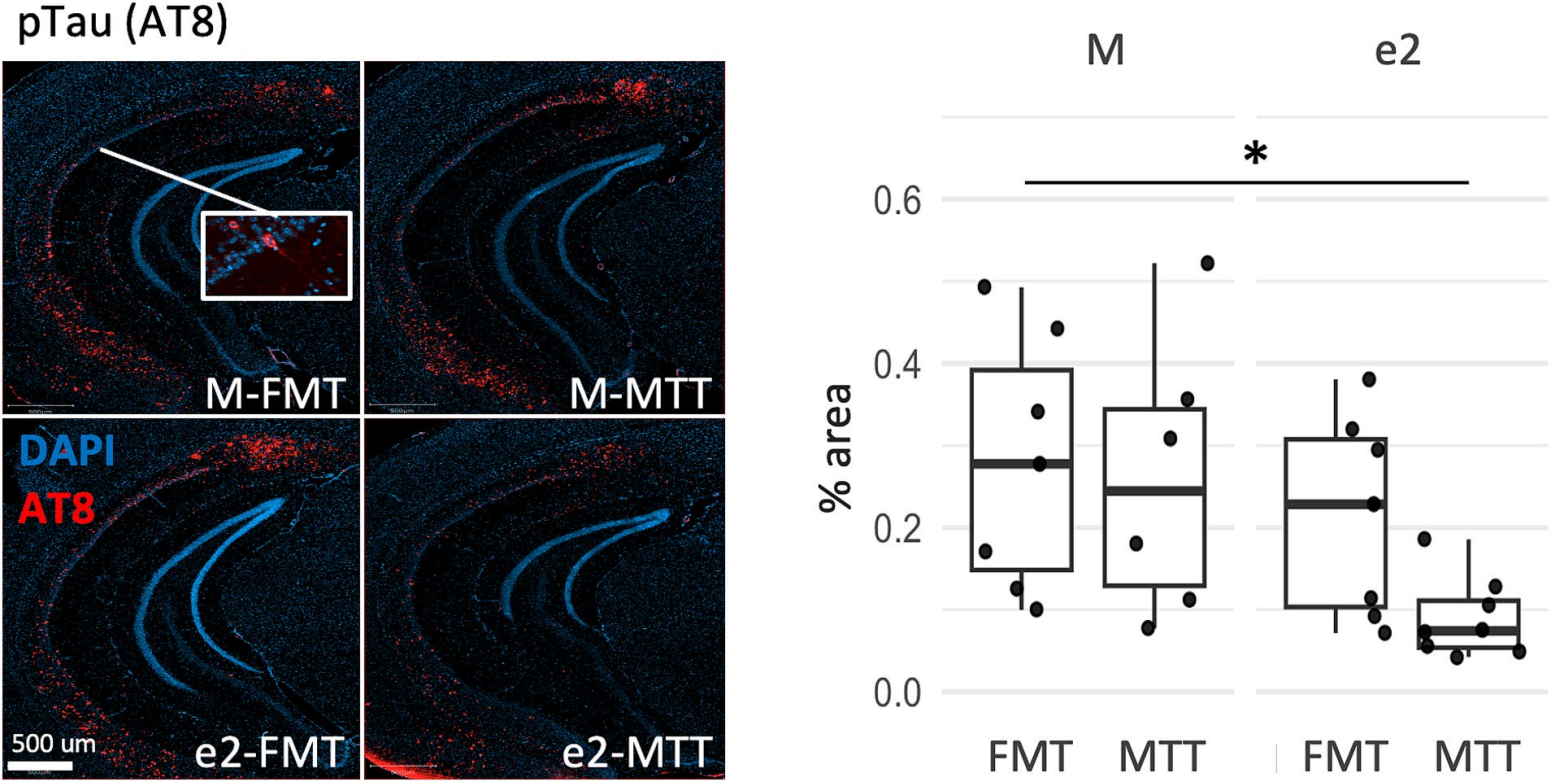
Effect of FMT from the APOEe2 carrier donor and MTT on decreasing pTau. Representative images of AT8 positive neurons and their percentage. Data are presented as boxplots with black horizontal lines indicating medians and circles indicating the subject’s values. Differences in means were tested by two-way ANOVA with donor (M or e2), transplantation protocol (FMT or MTT) and their interaction as factors and with Tukey test for multiple comparisons. *p < 0.05; **p < 0.01; ***p < 0.001. FMT, fecal microbiota transplantation alone; MTT, microbiota transfer therapy consisting of FMT plus antibiotics plus PPI treatment; e2, mice treated with bacteria isolated from an aged human APOEe2; M, mice treated with bacteria isolated from an untreated 3xTg mouse.

Tau pathology was also affected by the selection of the donor as a reduction of the percentage of AT8 positive neurons was observed in the e2-treated mice (*F* = 6.38, *p* = 0.018, Figure 3 and Supplementary Table 2). No significant effects were reported for Tx-soluble and Gua-insoluble pTau231 (*p* > 0.203; Supplementary Table 2).

### FMT from the APOEe2 donor increased whole hippocampal inflammation

**Figure 4:**
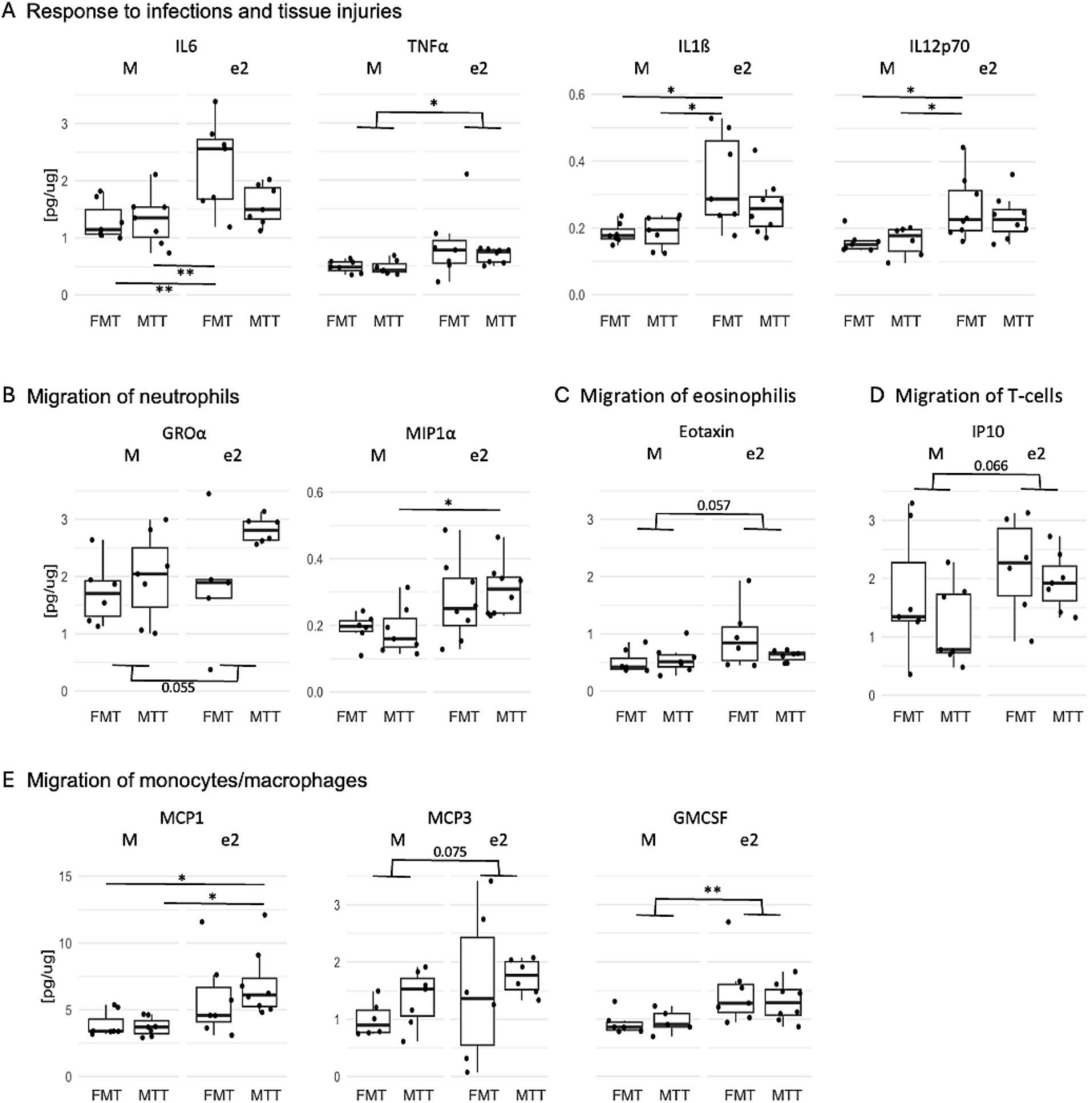
Effect of FMT from the APOEe2 carrier donor and MTT on increasing inflammatory mediators released in response to infections and tissue injuries **(A)**, involved in the regulation of migration of neutrophils **(B)**, eosinophilis **(C)**, T-cells **(D)** and monocytes/macrophages **(E)**. Protein quantification of inflammatory mediators was performed using ELISA. Data are presented as boxplots. Differences in means were tested by two-way ANOVA with donor (M or e2), transplantation protocol (FMT or MTT) and their interaction as factors and with Tukey test for multiple comparisons. *p < 0.05; **p < 0.01; ***p < 0.001. FMT, fecal microbiota transplantation alone; MTT, microbiota transfer therapy consisting of FMT plus antibiotics plus PPI treatment; e2, mice treated with bacteria isolated from an aged human APOEe2; M, mice treated with bacteria isolated from an untreated 3xTg mouse.

A MagPix ELISA test was conducted on cytokines and interleukins to assess the inflammatory status of the hippocampus. An effect of donor was observed for most of the inflammatory markers quantified (*F* > 6.14, *p* < 0.020; Figure 4 and Supplementary Table 2). In particular, mice treated with the bacteria from the e2 donor, regardless of the transplantation protocol, showed increased levels of molecules released in response to infections and tissue injuries (IL6, TNFα, IL1ß, IL12p70) as well as involved in the regulation of migration and infiltration of neutrophils (MIP1α, GROα), eosinophils (eotaxin), T-cells (IP10) and monocytes/macrophages (MCP1, GMCSF, MCP3) (Figures 4A–E, respectively).

### Effect of donor and transplantation protocol on the association of inflammation with amyloid and tau markers in the whole hippocampus

**Figure 5:**
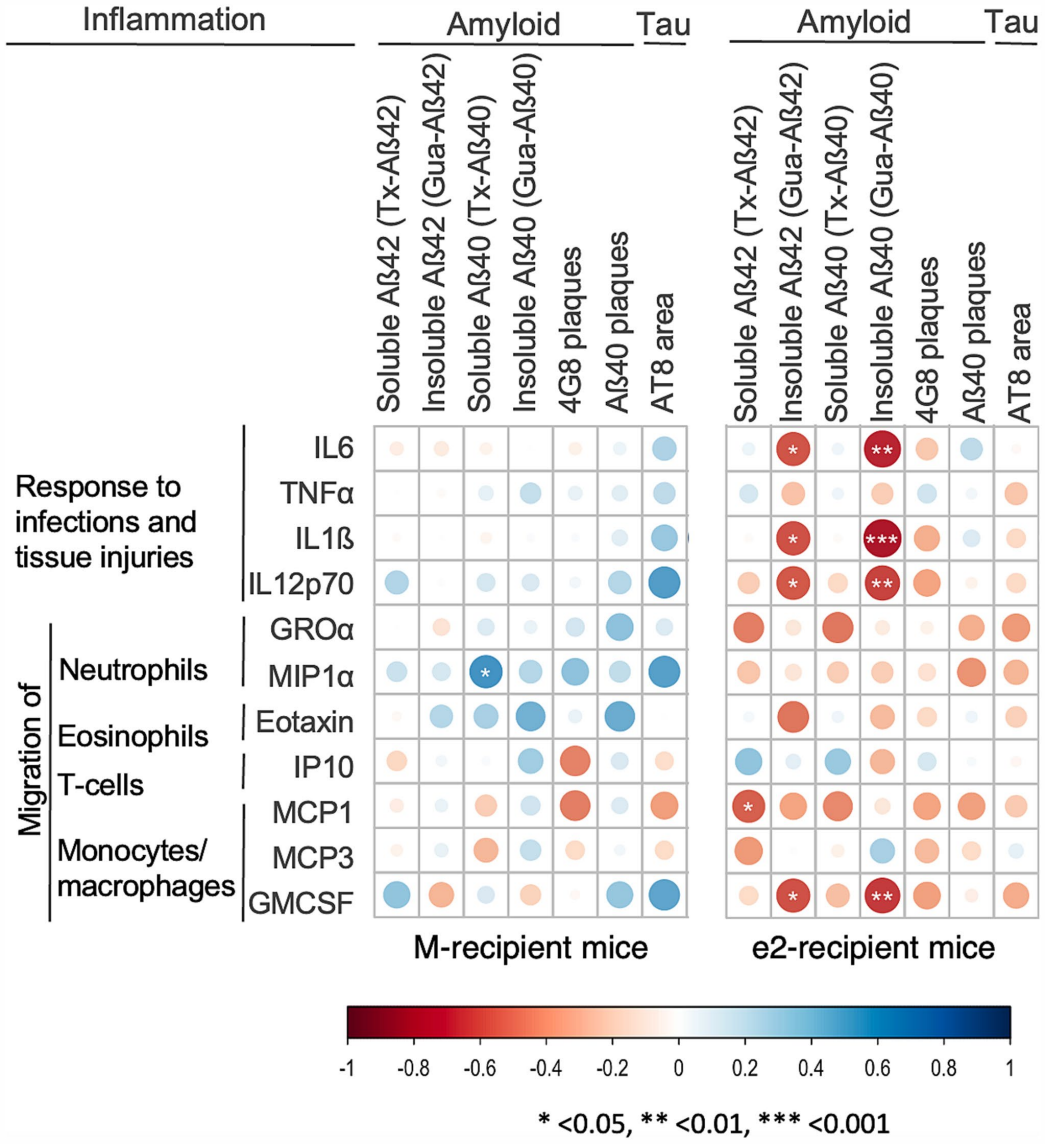
Inflammatory mediators correlated with low levels of amyloid and tau in e2 transplanted mice. Heatmap of the Spearman’s rho coefficient values (blue: positive; red: negative) indicating significant association (*p < 0.05; **p < 0.01; ***p < 0.001) in M-FMT and M-MTT pooled together (M-recipient mice) and e2-FMT and e2-MTT mice pooled together (e2-recipient mice). Correlation analyses included data for 9–14 mice for M-recipient mice, 11–16 mice for e2-recipient mice, depending on the combination of biomarkers considered.

Next, we performed Spearman correlation analyses of inflammatory mediators with amyloid and tau markers, focusing on those significantly affected by the donor or the transplantation protocol. Overall, the correlation heatmaps did not reveal any specific pattern of association in the M-treated mice (Figure 5, panel M-recipient mice). On the other hand, elevated levels of IL6, IL1ß, IL12p70, GMCSF were associated with low levels of highly aggregated Aβ42 and Aβ40 (−0.81 < rho<−0.62, *p* < 0.022) in the e2-FMT treated mice (Figure 5, panel e2-recipient mice). When e2-recipient mice were divided by transplantation protocol, these associations were weaker when MTT was administered in addition to FMT (−0.89 < rho<−0.77, *p* < 0.023 for e2-FMT; rho = −0.71, *p* < 0.047 for e2-MTT) (Supplementary Figure 2, panel e2-MTT). Finally, when split by transplant protocol, the association between high levels of inflammation and low levels of tau-positive neurons emerged in the e2-FMT group and not in e2-MTT (Supplementary Figure 2, panel e2-MTT).

## Discussion

This study tested the hypothesis that the protective effect of the e2 allele in AD could be mediated in part by the gut microbiota. Two types of transplantation protocols were tested, with simple FMT and in association with antibiotic and PPI treatment as in Kang et al. (2017). We analyzed the composition of the gut microbiota with 16SrRNA gene sequencing and identified an effect of the transplantation protocol per se on both alpha and beta diversity in line with previous report indicating the effect of both antibiotic treatment (Ramirez et al., 2020) and PPI usage (Zhang et al., 2023) in modulating the gut microbiota. The effect of the FMT donor (mouse or e2) on the microbiota composition was only detectable with the MTT protocol, confirming its role when transplanting human microbiome in mouse models. Interestingly, several genera of the Rikenellaceae family (*Rikenella* and *Rikenellaceae_RC9_gut_group*) were increased following e2-FMT as compared to mice when MMT was administered. This is in line with a recent meta-analysis, combining 11 studies, that identified a reduced abundance of Rikenellaceae family in patient with AD spectrum as compared to healthy controls (Hung et al., 2022). Bacteria belonging to this family showed a positive association with innate and a negative association with adaptive immune modulators in a cohort of healthy adults suggesting that specific microbial-immune system interactions might play a role in the maintenance of a functional intestinal barrier, and possibly to systemic (Riazati et al., 2023) and central immune activity.

We demonstrated that the transplantation of the bacteria isolated from an aged, amyloid negative, cognitively healthy APOEe2 carrier in the 3xTgAD mice ameliorated the neuropathological markers of AD pathology. Indeed, 3xTgAD mice transplanted with human e2 bacteria show reduction of both poorly and highly aggregated forms of amyloid, number of amyloid plaques and number of tau-positive neurons, compared with FMT from 3xTgAD mice. We found that e2 bacteria transplantation led to the greater hippocampal inflammation, which correlated with the reduction of amyloid and tau. These effects were observed in the absence of treatment with antibiotics and PPI, even though the latter improved some of the effects. To the best of our knowledge, this is the first study that performed FMT with bacteria from a cognitively healthy human donor as previous published reports used mouse donors (Elangovan et al., 2023; Sun et al., 2019) and, in one case, AD donors (Wang et al., 2022). Previous research in AD mouse models reported that amyloid pathology can be modulated by host microbiota but with contrasting results. Indeed, both a decrease (with a possible effect of the age of the recipient on efficacy) and an increase (Dodiya et al., 2021; Kim et al., 2020; Sun et al., 2019; Wang et al., 2022) in amyloid levels have been reported. We showed that amyloidosis in the brain is decreased in e2-recipient mice and that the different forms of amyloid do not appear to be identically sensitive to treatment. The forms recognized by anti-4G8 (amyloid and APP) showed a global effect of e2-FMT treatment, whereas the forms recognized by anti-Ab40 showed an effect amplified by MTT.

It is recognized that neuroinflammation can play a dual role in AD: can be protective by increasing Aβ and tau degradation and clearance but it can also contribute to their overproduction and induce neurodegeneration and synaptic loss (Novoa et al., 2022). Here, we showed that e2 treated mice reported hippocampal inflammation as indicated by the increased level of proteins involved in tissue repairing after injuries and in the recruitment of immune cells. In line, IL1ß, IL6, eotaxin, IP10 and MCP1 were found associated with amyloid deposits in the cortices of AD patients (Flores-Aguilar et al., 2021; Xia et al., 2000). Notably, previous studies have shown that APOEe2 carriers have significantly higher and APOEe4 carriers lower values of the pro-inflammatory C-reactive protein compared with APOEe3 carriers (Civeira-Marín et al., 2022; Hubacek et al., 2010; Schick et al., 2015). Data on the LPS-activation of primary astrocytes from APOE targeted replacement mice led to the same genotype-dependent differences in cytokine secretion (APOEe2 > APOEe3 > APOEe4) (Maezawa et al., 2006) suggesting that a possible biological substrate of the protective effect of APOEe2 is through modulation of inflammation. To date, the hippocampal inflammation in AD mouse model after microbiota transplantation has been little studied, and most existing data were obtained from antibiotic-treated mice re-colonized via FMT from non-treated mice (Dodiya et al., 2019, 2021). In general, these studies suggest that microglia, in the context of antibiotic treatment, may lose pro-inflammatory function and become more efficient at phagocytosis. In line, we found that MTT clearly weakened the associations of inflammatory proteins with amyloid and tau levels in e2 recipient mice. Moreover, both Aβ40 and tau-positive neurons decrease further when short term antibiotic treatment and PPI were administered, supporting this hypothesis of an increased phagocytic capacity of microglia. We speculate that in 3xTgAD mice, human e2 bacteria-and MTT treatment-induced perturbation of the microbiota may moderate the hippocampal innate immune response through distinct mechanisms, e2 activating cytokines and chemokines production and MTT inducing the microglia phagocytosis.

### Limitations and future directions

Although this study is the first one that tested the use of bacteria from a cognitively healthy human donor for 3xTg FMT, it has also limitations. The description of microglial morphology and functions are missing here, and an in-depth approach should be applied to dissect the impact of donor and MTT treatment on the microglia regulation. Here, we have described that a human APOEe2-derived microbiota transplantation led to a protective hippocampal pro-inflammatory status, associated with decreased AD pathology. However, our data have been generated by using a single human donor making it impossible to associate a single genetic variable (APOEe2) to causality. Future investigations with multiple donors and different animal models are required to generalize our findings. In addition, we and others reported that short chain fatty acids might have various effects on immune mediators as well as on cerebral amyloid deposition (Caetano-Silva et al., 2023; Erny et al., 2021; Marizzoni et al., 2023) but here the metabolic functions of the microbiota have not been analyzed. For M-FMT, feces of the last 24 h were collected from the cages and the anaerobic condition could not be maintained. Finally, behavioral assessment was not performed.

The importance of the microbiota in maintaining host health is now widely recognized. It has recently been shown that a healthy microbiota is necessary not only to maintain normal gut structure and function, but also to counteract aging-related changes, such as cognitive deficits, decreased neurogenesis (Grabrucker et al., 2023), and increased neurotoxicity due to exposure to environmental pollutants (Liu et al., 2024). Understanding the causative relation between microbiota and AD hallmarks will help to clarify the complex etiology of this insidious disease. This in turn will facilitate the development of products derived from gut bacteria (e.g., probiotic) for its prevention and treatment.

## Conclusion

Bacteria from a human APOEe2 donor reduced AD pathology and increased neuroinflammation suggesting that, (i) the gut microbiota may be a novel mediator of the protective effect of APOEe2 and, (ii) it might act through the activation of immune cells and the induction of cytokines and chemokines production. Although additional investigations are needed to further develop these findings, our results support the role of gut microbial dysbiosis in AD pathogenesis and suggest that targeting the gut microbiota may be a useful therapeutic strategy for the development of novel candidates for the AD treatment.

## Data Availability

The datasets presented in this study can be found in online repositories. The names of the repository/repositories and accession number(s) can be found below: https://www.ebi.ac.uk, PRJEB77863.

## Footnotes

^1^ https://www.arb-silva.de/
