# Supplementary material for: Stools from a human APOEe2 donor reduces amyloid and tau pathology and increases neuroinflammation in a 3xTg AD mouse model
Source: Front Aging Neurosci. 2025 Feb 14;17:1539067. doi: 10.3389/fnagi.2025.1539067 (PMC11868276; doi:10.3389/fnagi.2025.1539067)

Supplementary files

Additional file 1: Supplementary Information

Stools from a human APOEe2 donor reduces amyloid and tau pathology and increases neuroinflammation in a 3xTg AD mouse model

Moira Marizzoni[1]*, Benjamin B. Tournier[2]*, Claire Chevalier[3], Samantha Saleri[1], Aurélien Lathuilière[3], Kelly Ceyzériat[2,4], Arthur Paquis[3], Rahel Park[3], Emma Troesch[3], Annamaria Cattaneo[1,5], Philippe Millet[2], Giovanni B. Frisoni[3,6]

Supplementary Figures and Tables

Supplementary Table S1 is related to Figure 1.

Supplementary Table S2 is related to Figures 2, 3 and 4.

Supplementary Figure S1 is related to “FMT from the APOEe2 donor reduced amyloid and tau proteins in the whole hippocampus”.

Supplementary Figure S2 is related to Figure 5.

Supplementary table S1. Summary of the statistical results with the main effects of donor (M or e2), transplantation protocol (FMT or MTT) and their interaction, as derived from PERMANOVA analyses on gut microbiota (GM) β-diversity metrics.

Biomarker/ Metric	Donor	Transplantation protocol	Donor*Transplantation protocol
Bray-Curtis index	F=0.69; p=0.749	**F=2.30; p=0.018**	F=1.81; p=0.053
Jaccard index	F=1.34; p=0.178	**F=5.36; p<0.001**	F=1.60; p=0.083

Supplementary table S2. Summary of the statistical results with the main effects of donor (M or e2), transplantation protocol (FMT or MTT) and their interaction, as derived from 2-way ANOVA analyses on gut microbiota (GM) α-diversity metrics, amyloid (A), tau (T) and neuroinflammatory (I) data in the whole hippocampus.

	**Biomarker/ Metric**	**Donor**	**Transplantation protocol**	**Donor*Transplantation protocol**
GM	Shannon entropy	**F=4.89; p=0.036**	**F=8.53; p=0.008**	F=1.21; p=0.281
	Pielou's evenness	**F=4.33; p=0.048**	F=2.71; p=0.112	F=0.14; p=0.715
A	Soluble Aβ42 (Tx-Aß42)	**F=4.27; p=0.049**	F=1.96; p=0.174	F=0.00; p=0.996
	Insoluble Aβ42 (Gua-Aß42)	**F=7.92; p=0.009**	F=1.41; p=0.247	F=0.62; p=0.439
	Soluble Aβ40 (Tx-Aß40)	**F=4.62; p=0.042**	F=2.30; p=0.143	F=0.28; p=0.603
	Insoluble Aβ40 (Gua-Aß40)	**F=7.85; p=0.010**	F=0.27; p=0.610	F=1.08; p=0.209
	Aβ40 (N plaques)	**F=13.40; p=0.001**	**F=8.62; p=0.007**	F=1.13; p=0.298
	4G8 (N plaques)	**F=4.86; p=0.037**	F=0.67; p=0.422	F=0.79; p=0.282
	4G8 (% area)	F=1.98; p=0.172	F=1.09; p=0.307	F=0.48; p=0.493
	BACE1	F=2.50; p=0.127	F=0.19; p=0.664	F=2.90; p=0.102
	IDE	F=3.38; p=0.079	F=1.28; p=0.269	F=1.33; p=0.260
T	Soluble tau231 (Tx-tau231)	F=1.72; p=0.203	F=0.02; p=0.898	F=3.86; p=0.062
	Insoluble tau231 (Gua-tau231)	F=0.50; p=0.828	F=0.47; p=0.497	F=1.18; p=0.288
	AT8 (% area)	**F=6.38; p=0.019**	F=2.46; p=0.130	F=1.19; p=0.287
I	TSPO	F=0.37; p=0.548	F=0.23; p=0.637	F=0.35; p=0.562
	Eotaxin	F=4.01; p=0.057	F=1.29; p=0.268	F=2.67; p=0.116
	GMCSF	**F=8.41; p=0.008**	F=0.34; p=0.565	F=0.44; p=0.517
	GROalpha	F=4.06; p=0.055	F=0.38; p=0.545	F=0.73; p=0.402
	IL1beta	**F=12.73; p=0.001**	F=1.36; p=0.256	F=1.60; p=0.217
	IL6	**F=10.16; p=0.004**	*F=2.98; p=0.097*	*F=3.55; p=0.072*
	IL12p70	**F=12.12; p=0.012**	F=0.33; p=0.574	F=0.39; p=0.536
	IP10	F=3.71; p=0.064	F=1.58; p=0.222	F=0.20; p=0.662
	MCP1	**F=11.78; p=0.002**	F=0.40; p=0.531	F=0.70; p=0.412
	MCP3	F=3.48; p=0.075	F=0.01; p=0.966	F=0.44; p=0.516
	MIP1alpha	**F=10.43; p=0.003**	F=0.32; p=0.574	F=0.42; p=0.520
	TNFalpha	**F=6.14; p=0.020**	F=0.76; p=0.393	F=0.50; p=0.485

Supplementary Figure S1. Effect of donor selection and MTT on the enzymes involved in the amyloid synthesis (ß-site APP cleaving enzyme 1, BACE1) and degradation (insulin-degrading enzyme, IDE) in the whole hippocampus.

Differences in means were tested by two-way ANOVA with donor (M or e2), transplantation protocol (FMT or MTT) and their interaction as factors and with Tukey test for multiple comparisons. *, P< 0.05; **, P<0.01; ***, P< 0.001. Abbreviations: FMT, fecal microbiota transplantation alone; MTT, microbiota transfer therapy consisting of FMT plus antibiotics plus PPI treatment; e2, mice treated with bacteria isolated from an aged human APOEe2; M, mice treated with bacteria isolated from an untreated 3xTg mouse.

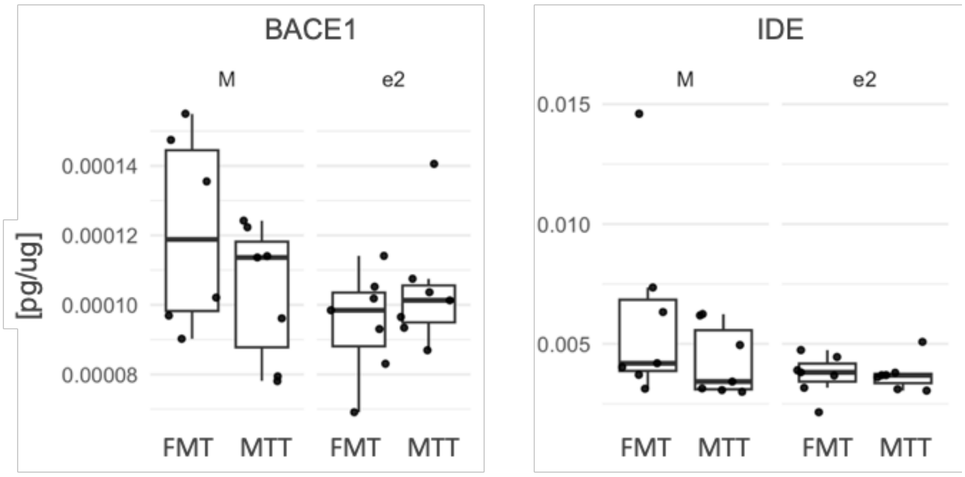

Supplementary Figure S2. Association of high levels of inflammatory mediators with low levels of amyloid and tau pathology in mice transplanted with bacteria from the APOEe2 carrier donor. Heatmap of the Spearman's rho coefficient values (blue: positive; red: negative) indicating significant association (*, P< 0.05; **, P< 0.01; ***, P< 0.001) in M-FMT, M-MTT, e2-FMT and e2-MTT mice. Correlation analyses included data for 5-7 mice for M-FMT, 4-7 mice for M-MTT, 5-8 mice for e2-FMT, 6-8 mice for e2-MTT, depending on the combination of biomarkers considered. Abbreviations: FMT, fecal microbiota transplantation alone; MTT, microbiota transfer therapy consisting of FMT plus antibiotics plus PPI treatment; e2, mice treated with bacteria isolated from an aged human APOEe2; M, mice treated with bacteria isolated from an untreated 3xTg mouse.

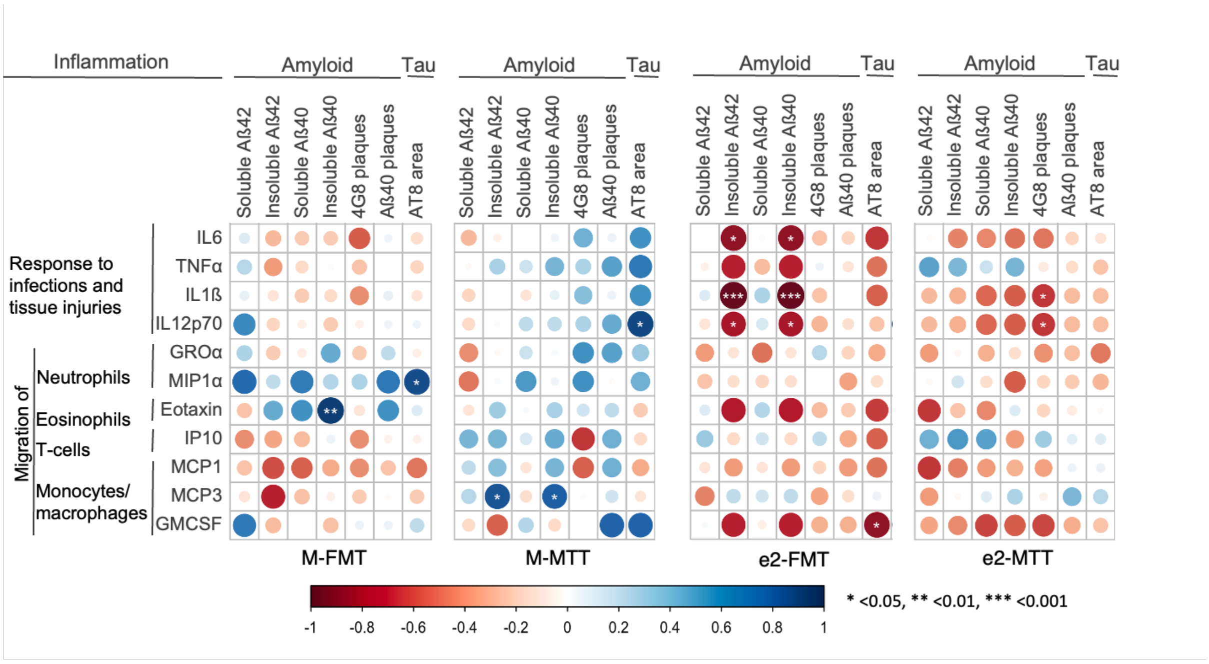

Supplement: Supplementary file 1 [file Data_Sheet_1.PDF]
